# Non-coding RNAs in the Ovarian Follicle

**DOI:** 10.3389/fgene.2017.00057

**Published:** 2017-05-12

**Authors:** Rosalia Battaglia, Maria E. Vento, Placido Borzì, Marco Ragusa, Davide Barbagallo, Desirée Arena, Michele Purrello, Cinzia Di Pietro

**Affiliations:** ^1^Section of Biology and Genetics G. Sichel, Department of Biomedical and Biotechnological Sciences, University of CataniaCatania, Italy; ^2^IVF Unit, Cannizzaro HospitalCatania, Italy

**Keywords:** human oocyte, ovarian follicle, follicular fluid, microRNAs, lncRNAs

## Abstract

The mammalian ovarian follicle is the complex reproductive unit comprising germ cell, somatic cells (Cumulus and Granulosa cells), and follicular fluid (FF): paracrine communication among the different cell types through FF ensures the development of a mature oocyte ready for fertilization. This paper is focused on non-coding RNAs in ovarian follicles and their predicted role in the pathways involved in oocyte growth and maturation. We determined the expression profiles of microRNAs in human oocytes and FF by high-throughput analysis and identified 267 microRNAs in FF and 176 in oocytes. Most of these were FF microRNAs, while 9 were oocyte specific. By bioinformatic analysis, independently performed on FF and oocyte microRNAs, we identified the most significant Biological Processes and the pathways regulated by their validated targets. We found many pathways shared between the two compartments and some specific for oocyte microRNAs. Moreover, we found 41 long non-coding RNAs able to interact with oocyte microRNAs and potentially involved in the regulation of folliculogenesis. These data are important in basic reproductive research and could also be useful for clinical applications. In fact, the characterization of non-coding RNAs in ovarian follicles could improve reproductive disease diagnosis, provide biomarkers of oocyte quality in Assisted Reproductive Treatment, and allow the development of therapies for infertility disorders.

## Introduction

Infertility, the inability to conceive and have children, is estimated to affect as many as 186 million people worldwide, and, consequently, represents an important social and medical problem (Inhorn and Patrizio, 2015). It can be considered a complex disease: first of all it is related to two different genomes (oocyte and sperm quality represent major determining factors in reproductive success), depends on endometrium receptivity and is influenced by several environmental factors (Koot and Macklon, 2013; Hart, 2016). In the same way as cancer, cardiovascular and neurodegenerative diseases, discovering a contributing factor and characterizing its involvement, is a difficult undertaking because the effect of any single factor may be obscured or confounded by other contributing factors (Manolio et al., 2009). To add to this complexity, male or female gametogenesis, fertilization and implantation, are regulated by different complex biological pathways and involve many molecules as mRNAs, non-coding RNAs, and proteins. In female gametogenesis, oocyte competence develops through protracted and complex processes beginning during embryonic life and ending at the moment that the MII oocyte is ovulated (Hutt and Albertini, 2007). During embryonic life, primordial germ cells (PGCs) migrate to the genital ridge, proliferate by mitosis transforming into primary oocytes. Primary oocytes enter in meiosis and become arrested in prophase I at the diplotene stage (dyctiate). At this time the oocyte is enclosed in a specialized lineage of ovarian somatic cells, pre-granulosa cells, to form a primordial follicle (Zuccotti et al., 2011). The primordial follicle pool, produced during embryonic life, represents the woman’s ovarian reserve (Reddy et al., 2010). After puberty, some primary follicles are cyclically recruited and develop through primary, secondary and antral stages. Inside antral follicles, in response to hormonal signaling, the oocytes are stimulated to resume meiosis. Most of the antral follicles undergo apoptosis, whereas only one, the dominant Graafian follicle, ovulates to release the mature egg, ready for fertilization (Sun et al., 2009; Zuccotti et al., 2011). At this stage, the follicles consist of the germ cell in the metaphase of the second meiotic division (MII oocytes), in a fluid-filled cavity called the antrum and different layers of somatic cells, the cumulus cells (CC) surrounding the oocytes and the granulosa cells (GC) as walls of follicle (Russell and Robker, 2007; Rodgers and Irving-Rodgers, 2010). Follicle development and oocyte maturation are strictly associated, in fact, the proliferation and the differentiation of somatic follicular cells occur in synchrony with the maturing oocyte, mediated by a constant exchange of signals between somatic cells and the germ cell (Russell and Robker, 2007). The cross-talk between the oocyte and somatic follicular cells occurs by gap-junctions established between the oocyte and CCs and through the Follicular Fluid (FF) accumulated inside the antrum (Rodgers and Irving-Rodgers, 2010). FF consists of a complex mixture of nucleic acids, proteins, metabolites, and ions, which are secreted by the oocytes and somatic cells (Revelli et al., 2009). Recently, it has been demonstrated that in human FF, microRNAs (miRNAs), carried by extracellular vesicles (EVs) such as microvesicles and exosomes are present (Santonocito et al., 2014). The discovery of mechanisms of autocrine and paracrine communication mediated by miRNAs, inside the ovarian follicle, has revealed that these non-coding RNAs represent important regulators inside the pathways involved in folliculogenesis and oocyte maturation (Santonocito et al., 2014; Di Pietro, 2016). Consequently, their characterization could improve our knowledge about female gametogenesis, and could pinpoint new molecules involved in reproductive disorders allowing the formulation of new therapeutic approaches.

The aim of this paper was to identify the miRNAs in the follicular microenvironment and position them within the different compartments of the ovarian follicle identifying the pathways regulated by their targets and the long non-coding RNAs (lncRNAs) possibly involved in oocyte growth and maturation.

## Materials and Methods

### Ethics Statement

The patients, included in IVF programs, signed an informed consent (in accordance with the Declaration of Helsinki) to participate in the research project, which comprised the use of collected FF and surplus MII oocytes. The study on human MII oocytes was approved by the Institutional Ethical Committee Catania 1.

### Sample Collection

Human oocytes and FF samples were collected from healthy women (without any ovarian pathology), ≤38 years old, undergoing intracytoplasmic sperm injections (ICSI) (Santonocito et al., 2014). FF of individual follicles was kept separated until decumulation of the oocytes to collect only the FF in which nuclear mature oocytes (metaphase II) had been identified. A total of 12 mature MII oocytes, two oocytes per woman, showing normal morphology, and 6 pools of FF were collected from individual follicles of 6 and 15 healthy women respectively, and used for miRNA expression profile analysis. The six couples of MII oocytes retrieved were separately placed in six independent Eppendorf tubes and rinsed in RNase-free water several times to remove any trace of cell culture medium. The oocytes were transferred to PCR tubes in 2 μl water and stored at -80°C before RNA extraction. Human FF samples were centrifuged for 20′ at 2,800 rpm at 4°C to remove follicular cell residue and any traces of blood; the supernatant was immediately transferred into a new Eppendorf tube and stored at -80°C for further analysis.

### RNA Isolation, Reverse Transcription and miRNA Profiling by Taqman Low Density Array

For fluids, miRNA isolation was performed by using Qiagen miRNeasy Mini Kit (Qiagen GmbH), according to the Qiagen Supplementary Protocol for the purification of small RNAs from serum and plasma and finally eluted in a 30 μl volume of RNAse-free water. Oocytes were incubated, after adding water (2 μl), for 1′ at 100°C according to previously published protocols with minor modifications (El Mouatassim et al., 1999; Di Pietro et al., 2008; Battaglia et al., 2016) in order to release nucleic acids. Samples (3 μl of total RNA from hFF and human MII oocytes), were retrotranscribed and preamplified. Amplified products were loaded onto microfluidic cards of the TaqMan Human MicroRNA Array A v2.0 (Applied Biosystems). To prepare the real time PCR reaction mix, 9 μl of undiluted pre-amplification product was added to 450 μl of 2X TaqMan Universal PCR Master mix, no AmpErase UNG (Applied Biosystems) and nuclease free water was added to a final volume of 900 μl. 100 μl of PCR reaction mix was loaded onto 384-well TaqMan Low Density Human MicroRNA array cards (TLDA). The qRT-PCR reaction was carried out according to the manufacturer’s instructions in a 7900HT Fast Real Time PCR System (Applied Biosystems).

### Analysis of miRNA Expression Data

miRNA expression profiles were analyzed using real-time RQ Manager software v1.2 (Applied Biosystems). For relative quantification of miRNAs we filtered miRNAs having Ct values below 37 and detected in all biological replicates. Statistically significant miRNA differences were identified by Significance of Microarrays Analysis (SAM)^1^, applying a two-class paired test among ΔCt of FF and oocytes samples by using a *p*-value based on 100 permutations; imputation engine: K-nearest neighbors, 10 neighbors; false discovery rate < 0.15 (Ragusa et al., 2014; Battaglia et al., 2016; Fendler et al., 2017). miRNA expression changes were calculated by applying the 2^-ΔΔCT^ method and using the average Ct of each plate as endogenous controls. We accepted only DE miRNAs common to least two SAM tests as reliable. Expression data in the Result section are shown as natural logarithms of relative quantity (RQ) values and the error was estimated by evaluating the 2^-ΔΔCt^ equation using ΔΔCt plus SD and ΔΔCt minus SD (Livak and Schmittgen, 2001). The expression data have been deposited in NCBI’s Gene Expression Omnibus (Edgar et al., 2002) and are accessible through GEO Series accession number GSE98103.^2^

### miRNA Target Prediction, Go and Pathway Analysis

In order to gain insights into biological processes (BP) regulated by FF and oocyte miRNAs we retrieved validated targets by using miRTarBase v6.0^3^ to select targets experimentally verified in humans with strong validation methods (Reporter assay, Western blot and RT-qPCR). Afterward, we explored miRNA target expression through the comparison with protein coding genes expressed in ovarian follicles in humans, available from OKdb^4^, and then we analyzed their Gene Ontologies (GO) and Pathways by the Panther classification system v10.0.^5^ The statistical overrepresentation test was executed and the Bonferroni correction for multiple testing was used to correct the *P*-value. GOs and molecular pathways with a *P*-value < 0.05 were chosen. Target genes of miRNAs commonly expressed in hFF and oocytes were subsequently used in a signaling pathway enrichment analysis in Diana-miRPath v3.0.^6^ The list of genes expressed in the human ovarian follicle was imported into Diana-miRPath to carry out pathway analysis of experimentally validated miRNA gene targets, according to the Kyoto Encyclopedia of Genes and Genomes (KEGG). The FDR method was implemented to select the biological pathways with a threshold of significance defined by *P* < 0.05 and a microT threshold of 0.8.

### Prediction of lncRNAs Implicated in miRNA Regulation

In order to explore whether lncRNAs might have a regulatory function in oocyte maturation and early embryo development, we searched for experimentally verified miRNA-lncRNA interactions on LncBase v.2^7^ (Paraskevopoulou et al., 2016). The identification of miRNAs that are interacting with lncRNAs was performed by selecting those experimentally verified in homo sapiens (by Northern Blot, luciferase reporter assay and qPCR), and found expressed in the ovary, embryo and ESCs, with a prediction score above the 75th percentile. To have a better understanding of lncRNA functions in human oocytes we drew interactions among candidate molecules using information from literature data and NPInter v3.0^8^.

## Results

### Profiling of microRNAs in Ovarian Follicle Compartments

**FIGURE 1:**
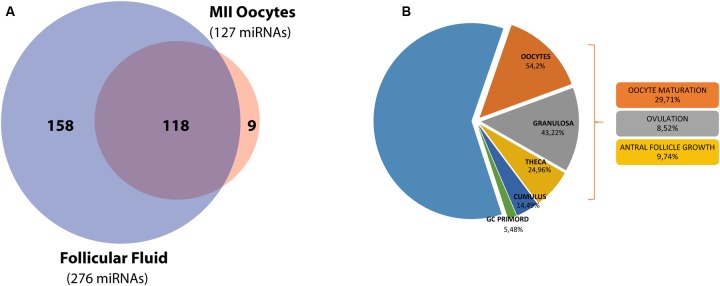
**miRNA distribution in the ovarian follicle and functional analysis of the target genes. (A)** Venn diagram shows the overlap between miRNA sets in hFF and the mature MII oocyte. **(B)** A second diagram shows miRNA target expression and function inside the ovarian follicle.

Using TaqMan Low Density Array (TLDA) technology, we determined the expression profile of 384 miRNAs on 6 pools of FF and 6 pools of MII oocytes from healthy women. We identified 267 miRNAs in FF and 176 in oocytes. In order to characterize the miRNA content in the respective follicle components, we compared the sets of identified miRNAs and found 118 miRNAs (Common-miRNAs), including small nuclear RNA U6, in both components (**Figure 1A**). Moreover, by qualitative analysis, we detected a subsets of miRNAs specifically expressed in FF that were undetected in oocytes, and *vice versa*. In particular, we identified 158 miRNAs only in the FF compartment (FF-miRNAs), whereas we found 9 miRNAs exclusively expressed in human MII oocytes (O-miRNAs) (**Figure 1A**). In order to explore their role in ovarian follicle maturation, we compared the validated targets of the identified miRNAs with protein coding genes expressed in the human ovary. Of the 2,067 target genes ∼39% were known to be expressed within different follicle cell types and involved in different aspects of follicle development: oocyte maturation (29.71%), ovulation (8.52%) and antral follicle growth (9.74%) (**Figure 1B**). Moreover, we identified 277 miRNAs (FF-miRNAs and Common-miRNAs) with an overlap of 82% with miRNAs annotated on web-based resource ExoCarta. On the other hand, miR-515-5p, miR-519c-3p, miR-520d-5p miR-548a-3p, and miR-548c-3p, specifically expressed in oocyte, are not found incorporated in exosome vesicles.

### Gene Ontology and Pathway Analysis of FF-miRNAs and O-miRNAs

**FIGURE 2:**
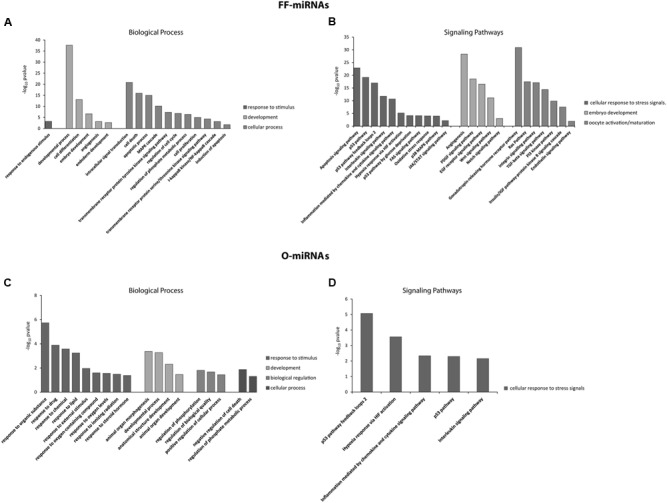
**Significant overrepresentation of GOs, in terms of Biological Processes, and signaling pathways for miRNAs identified in hFF (FF-miRNAs) and the MII oocytes (O-miRNAs) are shown in (A,B)** and **(C,D)**, respectively. The significance values are reported as -log10 (*P*-value).

Gene ontologies and Pathway analysis were independently performed on FF-miRNA and O-miRNA validated target genes. According to the number of miRNAs identified, the number of mRNA targets is quite different in the two compartments (1,099 FF-miRNA targets and 19 O-miRNA targets): for this reason the data cannot be considered all together. The most significant BP involving the targets of both FF-miRNAs and O-miRNAs are related to cellular response to stimulus, development and the regulation of cellular processes (**Figures 2A,C**). FF-miRNAs were statistically more represented in developmental processes, cell differentiation, intracellular signal transduction, cell death, apoptotic processes and the response to endogenous stimuli than O-miRNAs (**Figures 2A,C**). Conversely, O-miRNAs showed a significant enrichment of target genes in GO terms related to the response to organic substances, drugs, chemicals, lipids, external stimuli, oxygen containing compounds and other processes such as organ morphogenesis, developmental processes, regulation of biological quality and negative regulation of cell death (**Figures 2A,C**). The most significant pathways are regulated by both FF-miRNAs and O-miRNAs and are associated with the cellular response to stress signals, such as p53 feedback loops 2, p53, Hypoxia response via HIF activation, inflammation mediated by chemokines and cytokines, and interleukin signaling pathway (**Figures 2B,D**).

### Quantification and Pathway Analysis of Common-miRNAs

**FIGURE 3:**
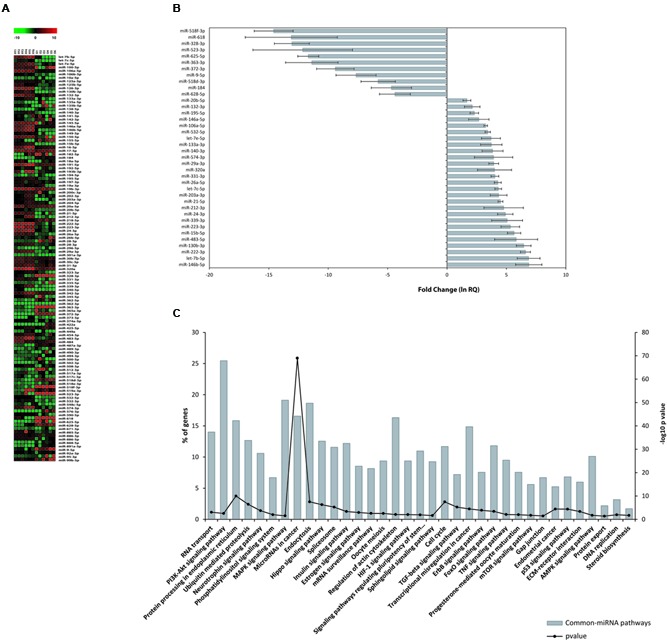
**miRNA expression in hFF and human oocytes. (A)** Heat map of normalized miRNA expression data (–DCT values) of 118 Common-miRNAs for hFF and oocyte samples. The red and green colors represent up and down regulated miRNA expression levels, respectively. Equally expressed miRNAs are indicated in black. **(B)** Relative expression levels of 27 and 11 miRNAs that were differentially expressed between FF and oocytes. **(C)** Signaling pathway enrichment analysis for common miRNAs with KEGG against listed target genes. The probability values are reported as –log10 (*P*-value).

Finally, because it is not possible to establish the specific cell type from which the Common-miRNAs originate, we compared the expression profiles of 118 miRNAs, co-expressed in FF and oocytes. The heat map diagram in **Figure 3A** shows miRNA normalized expression levels across the different sample types (**Figure 3A**). SAM analysis revealed 38 miRNAs displaying statistical significant differences between human FF and MII oocytes (**Figure 3B** and **Supplementary Table S1**). Approximately 16% of the Common-miRNAs showed no significant variation between the two ovarian follicle components. On the contrary, we found 27 miRNAs significantly up-regulated in human FF (with respect to oocytes). Interestingly, let-7b-5p, miR-15b-5p, miR-24-3p, miR-130b-3p, miR-146b-5p, miR-212-3p, miR-222-3p, miR-223-3p, miR-339-3p and miR-483-5p showed expression fold changes higher than 100-fold (ln RQ > 4.7) compared to oocytes (**Figure 3B** and **Supplementary Table S1**). In contrast, 11 miRNAs (miR-9-5p, miR-184, miR-328-3p, miR-363-3p, miR-372-3p, miR-518d-3p, miR-518f-3p miR-523-3p, miR-618, miR-625-5p, and miR-628-5p) were significantly up-regulated in human oocytes (with respect to FF) (**Figure 3B** and **Supplementary Table S1**). Finally, Common-miRNAs, analyzed by KEGG analysis, showed their involvement in regulating 48.9% of genes expressed in the human ovarian follicle. Most of the significant pathways are shared with FF-miRNAs but we also found many pathways involved in oocyte maturation, regulation of pluripotency in stem cells and miRNAs in cancer. Moreover, protein processing in the endoplasmic reticulum, endocytosis, gap junction and protein export are also well represented (**Figure 3C**).

### Long Non-coding RNAs in Oocytes

**FIGURE 4:**
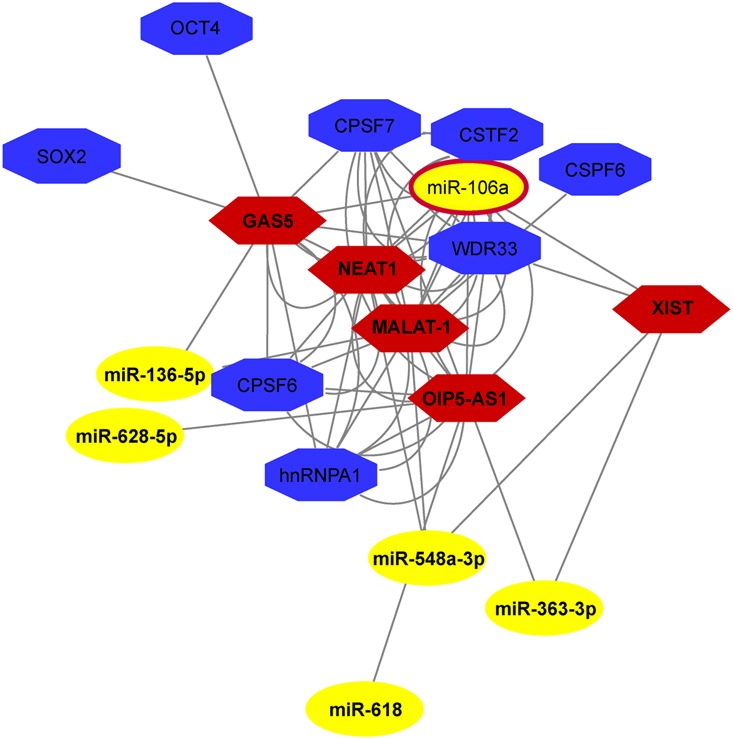
**ncRNA network in human MII oocytes.** The Network, drawn by NPInter v3.0, shows the interaction among lncRNAs and miRNAs involved in stemness, RNA maturation and epigenetics.

Prediction of experimentally verified interactions of lncRNAs with the 16 O-miRNAs was implemented by DIANA-LncBase database. A total of 41 lncRNAs were significantly associated with 9 O-miRNAs (**Table 1**). Each of them is expressed in embryonic cells, in the ovary or in the placenta and are involved in several human pathologies. We found that 17 lncRNAs were annotated as long intergenic RNAs (lincRNAs), 9 as antisense transcripts of genes, 6 as transcript isomorphs, 5 as retained introns, 4 as sense transcripts and 11 as circular RNAs (circRNAs) in the database circBase^9^ (**Table 1**). Network analysis showed the interactions among lncRNAs and miRNAs involved in stemness, RNA maturation and epigenetics (**Figure 4**).

**Table 1:** LncRNAs in human oocytes.

miRNA	lncRNA	Prediction score	Biotype	Chromosome
miR-9-5p	CTB-89H12.4	0.994	Retained_intron^∗^	5
	TUG1	0.985	Antisense^∗^	22
	RP11-793H13.8	0.962	Retained_intron	12
	SNHG14	0.934	Antisense^∗^	15
	RP11-314B1.2	0.930	lincRNA	2
	RP11-383J24.6	0.868	lincRNA	8
	RP11-436K8.1	0.858	lincRNA	1
	TMEM256-PLSCR3	0.857	Sense	17
	CTD-2368P22.1	0.808	Retained_intron	19
	AC007246.3	0.791	Antisense	2
	XLOC_000918	0.789	Transcript isomorph	1
	RNF144A-AS1	0.780	Antisense	2
	RP4-717I23.3	0.779	lincRNA^∗^	1
	IPO11-LRRC70	0.777	Sense	5
	XLOC_008152	0.771	Transcript isomorph	X
	AC093323.3	0.759	lincRNA	4
	DPP10-AS1	0.755	Antisense	2
miR-136-5p	MALAT-1	0.937	lincRNA^∗^	11
	GAS5	0.903	Retained_intron^∗^	1
	RP11-383J24.6	0.802	lincRNA	8
	RP3-468B3.2	0.781	lincRNA	6
miR-363-3p	XIST	0.919	lincRNA^∗^	X
	XLOC_008152	0.914	Transcript isomorph	X
	RP11-67L2.2	0.797	lincRNA	3
	OIP5-AS1	0.773	Antisense^∗^	15
	CASC7	0.769	lincRNA	8
	CTD-3099C6.9	0.751	Sense_intronic	19
miR-519c-3p	CTB-89H12.4	0.990	Retained_intron^∗^	5
	LOC388692	0.871	lincRNA	1
	GABPB1-AS1	0.792	Antisense	15
miR-520d-5p	CTB-89H12.4	0.974	Retained_intron^∗^	5
	CASC7	0.935	lincRNA	8
	NORAD	0.891	lincRNA	20
miR-548a-3p	CASC7	0.960	lincRNA	8
	CTB-89H12.4	0.890	Retained_intron^∗^	5
	LINC01355	0.944	lincRNA	1
	MALAT-1	0.865	lincRNA^∗^	11
	NEAT1	0.950	lincRNA	11
	RP6-24A23.7	0.761	Sense-overlapping	X
	XIST	0.933	lincRNA^∗^	X
	XLOC_006828	0.776	Transcript isomorph	8
	XLOC_011568	0.770	Transcript isomorph	15
	ZFAS1	0.761	Antisense^∗^	20
miR-618	NORAD	0.857	lincRNA	20
	OIP5-AS1	0.884	Antisense^∗^	15
	SNHG1	0.753	Retained_intron ^∗^	11
	SNHG14	0.770	Antisense^∗^	15
	SNORD116-20	0.894	lincRNA ^∗^	15
miR-625-5p	CASC7	0.900	lincRNA	8
	KMT2E-AS1	0.834	lincRNA	7
	SRRM2-AS1	0.794	Antisense	16
	CTD-2619J13.14	0.769	lincRNA	19
	XLOC_012097	0.752	Transcript isomorph	17
miR-628-5p	OIP5-AS1	0.769	Antisense^∗^	15

*Diana-LncBase prediction of miRNA-lncRNA interactions. ^∗^Indicates lncRNAs annotated as circRNA in the database circBase (www.circbase.org).*

## Discussion

Regulatory non-coding RNAs (ncRNAs) control different points of gene expression including chromatin architecture, epigenetics, transcription, RNA splicing, editing, translation and turnover. They are involved in every physiological process and consequently their sequences or expression alterations cause or contribute to different human diseases (Bartel, 2004; Croce, 2009; Fu, 2014). RNA regulatory networks include miRNAs, other classes of small regulatory RNAs and thousands of longer transcripts, named long non-coding RNAs that can be categorized in sense, antisense, bidirectional, intronic, and intergenic transcripts. Surely, the miRNA world is becoming increasingly well known to researchers, but for lncRNAs most of their functions are unknown, even if different papers have demonstrated their role in cell physiology and human pathologies. Their presence in biological fluids, as well as their expression profiles associated with specific human phenotypes, open up the possibility of using them, especially miRNAs, as molecular markers of human diseases (Weber et al., 2010; Cortez et al., 2011). Moreover, their presence in EVs, identifying miRNAs as molecular tools of communication among different cells, gives the possibility to plan specific and individualized therapies (Cortez et al., 2011; Kosaka et al., 2013; Matsui and Corey, 2016).

In reproductive biology, throughout the last decade, the role of miRNAs emerged in an important way and different studies attempted to associate specific miRNA expression profiles to oocyte quality, in granulose and cumulus cells and in FF (Li et al., 2015; McGinnis et al., 2015). Hence, great efforts were made to find promising molecular markers, in order to select the best oocytes to use in In Vitro Fertilization protocols and provide possible therapies to improve oocytes quality (Li et al., 2015; McGinnis et al., 2015).

Unfortunately, the ovarian follicle constitutes, to date, a difficult model to study for different reasons. It is made up of different cell types and represents the functional unit that ensures proper oocyte maturation by processes that begin during the embryonic stage and continue during a woman’s life until ovulation (Hutt and Albertini, 2007). The mature ovarian follicle and ovulation represent the results of different molecular processes prolonged in time and influenced by genetic background, environment and a woman’s life style. To analyze the single components of this complex unit, at the final stage, (MII oocyte, granulosa or cumulus cells, FF) does not provide all the information we need to fully understand the oogenesis and associate specific markers to specific phenotypes. Moreover, in humans, it is not possible to perform functional studies because of ethical limits.

The aim of this paper was to investigate the role of miRNAs and lncRNAs in human ovarian follicles, trying to establish, as far as possible, their potential role within the different components of the follicle.

Firstly, we identified 285 miRNAs inside human ovarian follicles (**Figure 1**). Interestingly, about 39% of their validated targets are expressed in different cellular components of the ovarian follicle; they are abundant in oocytes (54.2%) and predominantly involved in oocyte maturation (29.71%). 118 miRNAs (more than 40%) were shared by FF and the mature MII oocyte, while FF-miRNAs fraction was larger than the miRNAs exclusively found in oocytes. We propose that the 158 miRNAs absent in MII oocytes and exclusively present in FF have been transcribed by somatic follicular cells. Subsequently secreted in FF, these miRNAs could act as paracrine factors for the different somatic cells and regulate follicular growth. As expected, 82% of FF-miRNAs have been described in exosomes, in fact, according to previous studies, specific miRNAs are preferentially sorted into vesicles (Batagov et al., 2011). On the other hand the 9 miRNAs, absent in FF and exclusively present in the oocyte (O-miRNAs), could represent maternal RNAs, that the germ cells accumulate during their differentiation. In fact, during its growth the oocyte transcribes and stores mRNAs, miRNAs and probably long non-coding RNAs to use them during the first phase of development, before the activation of the embryo genome (Schier, 2007). Interestingly, we found miR-515-5p, miR-519c-3p, miR-520d-5p, miR-548a-3p, and miR-548c-3p specifically expressed in oocytes, and according to ExoCarta, these have not been found incorporated in exosome vesicles. Moreover, miR-515-5p, miR-519c-3p and miR-520d-5p, members of C19MC, which is a primate-specific cluster, seem to have a role in early embryo development during maternal-zygotic transition, when zygotic transcription starts and maternal mRNAs are degraded (Donker et al., 2012; Battaglia et al., 2016). Go analysis showed that development processes, regulation of cell cycle, signal transduction, and cell death represent BPs shared by oocytes and somatic follicular cells. In the same way, the p53 pathway is significant in both cell types. This confirmed our knowledge about the processes involved in oocyte maturation and carried out by both compartments (Hutt and Albertini, 2007). Apoptosis has been amply described in the mammalian ovary (Tilly, 2001). Cell death by apoptosis affects about 99% of primordial follicles present at birth in mammalian ovaries (Tiwari et al., 2015). The production of the mature oocyte ready for fertilization is a highly selective process: only a few follicles in the human ovary survive to complete their growth, only fully competent oocytes will be ovulated and only an embryo without major genetic alterations will be capable of uterine implantation. Apoptosis of granulosa cells reduces the number of recruited follicles and apoptotic machinery in MII oocytes selects viable embryos (Tilly, 2001; Santonocito et al., 2014). Interestingly, we found different BPs involved in the response to exogenous stimuli, significant only for O-miRNAs (**Figure 2C**). Among the different cells of the ovarian follicle the germ cell is unique: it must be able to respond and react to external stimuli more efficiently than somatic cells. It has been described that the maturing oocyte and early embryo are quite sensitive to exogenous stresses. Oocytes and early embryos can undergo physiological adaptations to environmental perturbations; these adaptations could influence the embryo genome signature involving epigenetic modification (Latham, 2015).

As concerns the 118 shared miRNAs, not being able to pinpoint the cells producing them, we can assert that these miRNAs could mediate the communication between the oocyte and somatic cells. Eleven of them, more abundant in oocytes, could have been transcribed by germ cells and used as signaling for cumulus and granulose cells. This hypothesis is supported by the finding that miR-518d-3p, miR-518f-3p, and miR-523-3p, up-regulated in oocytes, are members of the C19MC cluster, in the same way of some O-miRNAs. These miRNAs could be transcribed together, some of them stored in oocytes, others packaged in vesicles and secreted in FF. A similar consideration can be made for miR-372 (up-regulated in oocytes) and miR-371 (O-miRNA). On the contrary, the miRNAs up-regulated in FF, especially let-7b-5p, miR-15b-5p, miR-24-3p, miR-130b-3p, miR-146b-5p, miR-212-3p, miR-222-3p, miR-223-3p, miR-339-3p and miR-483-5p, with fold change values higher than 100-fold, could be transcribed in somatic follicular cells and move to oocytes by means of exosomes. Enrichment analysis of validated target genes of Common-miRNAs showed a strong correlation with the maintenance of the primordial follicle quiescent stage, oocyte maturation, oocyte meiosis, development, cancer and stem cell related pathways. Moreover, a high representation of apoptosis signaling pathway, hypoxia response via HIF activation and oxidative stress response have been detected (**Figure 3C**).

To understand miRNA-mediated gene regulation, we investigated if other classes of non-coding RNAs play a role inside the ovarian follicle. LncRNAs can act as miRNA sponges, reducing their regulatory effect on mRNAs introducing an extra layer of complexity in the miRNA-target interaction network (Paraskevopoulou and Hatzigeorgiou, 2016). Moreover, the important role of lncRNAs in chromatin remodeling is well known, as well as the importance of this process in oocyte maturation, when, before meiosis resumption, transcriptional silencing is mediated, over all, by mechanisms involved in large-scale chromatin structure changes. The cellular and molecular pathways involved in these processes are today poorly understood and lncRNAs could play a major role (De La Fuente et al., 2004). Recently, lncRNAs were identified in granulosa and cumulus cells, oocytes and early embryos, and their role in oocyte and early embryo development has been suggested (Yan et al., 2013; Yerushalmi et al., 2014; Hamazaki et al., 2015; Xu et al., 2015). A comprehensive review on lncRNA functions in mammalian and in different species has been recently published (Taylor et al., 2015).

By using bioinformatic prediction we found 41 lncRNAs significantly correlated with 9 oocyte miRNAs (**Table 1**). Most of them are expressed in embryonic cells, in the ovary or in the placenta and are involved in several human pathologies. NEAT1 was found exclusively localized in paraspeckles and is a core component of these nuclear bodies involved in nuclear retention of mRNAs (Bond and Fox, 2009). Moreover, NEAT1 seems to be essential for the formation of the corpus luteum and the establishment of pregnancy in mice, although its precise molecular mechanism remains to be investigated (Nakagawa et al., 2014). Recently, increased levels of NEAT1 was found associated with placental dysfunction in Idiopathic Intrauterine Growth Restriction (IUGR) fetuses (Gremlich et al., 2014). Another lincRNA that was found to reside predominantly in the nucleus is the Metastasis associated lung adenocarcinoma transcript-1 (MALAT-1) that localizes to nuclear bodies known as nuclear *speckles* involved in pre-mRNA splicing (Lennox and Behlke, 2016). MALAT-1 down-regulation was described to impair proliferation, cell cycle, apoptosis, and migration of trophoblast cells involved in the preeclampsia (Chen et al., 2015). Another human lincRNA, the non-coding RNA activated by DNA damage (NORAD) is induced after DNA damage in a p53-dependent manner and plays a crucial role in maintaining genomic stability by sequestering PUMILIO proteins, which repress the stability and translation of mRNAs to which they bind (Lee et al., 2016). In view of the location and the function of these 3 lncRNAs in regulation of mRNA stability and translation, we suppose that in oocytes these could stabilize maternal RNAs, allowing their storage and use during early development, before the activation of embryo genome, as it has been described for cytoplasmic polyadenylation (Reyes and Ross, 2016). Not surprisingly, we found the X-inactive specific transcript (XIST) and the growth arrest specific 5 (GAS5), two lncRNAs involved in embryogenesis. It has been demonstrated that many aspects of embryogenesis seem to be controlled by ncRNAs, including the maternal–zygotic transition, the maintenance of pluripotency, the patterning of the body axes, the specification and differentiation of cell types and the morphogenesis of organs (Pauli et al., 2011). XIST, responsible for the mammalian X chromosome inactivation, is the first lncRNA expressed starting at the 4-cell stage of human preimplantation embryos, consistent with embryonic genome activation, and iPSC reprogramming (Briggs et al., 2015).

Encoded within introns GAS5 increases OCT4, NANOG and SOX2 by Nodal regulation and is directly regulated by these stemness factors in hESCs forming a circuit that promotes pluripotency (Xu et al., 2016). OIP5-AS1 is an antisense transcript of the Opa interacting protein 5 (OIP5) gene. The protein encoded by this gene localizes to centromeres, where it is essential for recruitment of CENP-A and it is required for centromeric heterochromatin organization. Expression of this gene is upregulated in several cancers, making it a putative therapeutic target.

Recently, a new regulatory circuitry in which RNAs can crosstalk with each other and modulate the biological function of miRNAs has been proposed (Cesana et al., 2011). LncRNAs that localize primarily in the nucleus (e.g., XIST, NEAT1, MALAT-1) have been described to physically interact with mature miRNAs (Leucci et al., 2013; Gernapudi et al., 2015; Yu et al., 2017). Several observations have shown the presence of mature miRNAs in the nucleus (Hwang et al., 2007; Rasko and Wong, 2017). In facts, miRNAs can be transported from the cytoplasm to the nucleus and act in an unconventional manner to regulate the biogenesis and functions of ncRNAs (Liang et al., 2013).

Among the identified lncRNAs in oocytes, NEAT1, MALAT-1, GAS5, XIST and OIP5-AS1 have been predicted as components of the same network (**Figure 4**). Even if the mechanisms of most of these lncRNAs remain unknown, and it remains to be seen whether they can function within human ovarian follicle, these putative interactions lead us to hypothesize a possible role inside the female human germ cell.

## Conclusion

Understanding the regulation of gene expression inside the ovarian follicle is important in basic reproductive research and could also be useful for clinical applications. In fact, the characterization of non-coding RNAs in ovarian follicles could improve reproductive disease diagnosis, provide biomarkers of oocyte quality in Assisted Reproductive Treatment, and develop therapies for infertility disorders.

## Author Contributions

CDP conceived and designed the study. RB performed the experiments. CDP and RB analyzed, interpreted the data and wrote the manuscript. DA contributed to experiments and the bioinformatics analysis. MV and PB participated in sample collection. MR and DB contributed to the analysis of data. MP contributed to the critical revision of the manuscript.

## Conflict of Interest Statement

The authors declare that the research was conducted in the absence of any commercial or financial relationships that could be construed as a potential conflict of interest.

## Footnotes

1 http://www.tm4.org

2 https://www.ncbi.nlm.nih.gov/geo/query/acc.cgi?acc=GSE98103

3 http://mirtarbase.mbc.nctu.edu.tw/

4 http://okdb.appliedbioinfo.net/

5 http://pantherdb.org

6 http://snf-515788.vm.okeanos.grnet.gr/

7 http://www.microrna.gr/LncBase

8 http://www.bioinfo.org/NPInter/

9 http://www.circbase.org

## Supplementary Material

TABLE S1 **Differentially expressed (DE) miRNAs from qRT-PCR comparative analysis between human follicular fluid (hFF) and human mature MII oocytes**.
